# Autophagic and endo-lysosomal dysfunction in neurodegenerative disease

**DOI:** 10.1186/s13041-019-0504-x

**Published:** 2019-11-29

**Authors:** Bilal R. Malik, Daniel C. Maddison, Gaynor A. Smith, Owen M. Peters

**Affiliations:** 1 UK Dementia Research Institute at Cardiff University, Cardiff, Wales UK; 2 School of Biosciences, Cardiff University, Cardiff, Wales UK; 3 School of Medicine, Cardiff University, Cardiff, Wales UK

**Keywords:** Autophagy, Endo-lysosome, Mitophagy, Neurodegeneration

## Abstract

Due to their post-mitotic state, metabolic demands and often large polarised morphology, the function and survival of neurons is dependent on an efficient cellular waste clearance system both for generation of materials for metabolic processes and removal of toxic components. It is not surprising therefore that deficits in protein clearance can tip the balance between neuronal health and death. Here we discuss how autophagy and lysosome-mediated degradation pathways are disrupted in several neurological disorders. Both genetic and cell biological evidence show the diversity and complexity of vesicular clearance dysregulation in cells, and together may ultimately suggest a unified mechanism for neuronal demise in degenerative conditions. Causative and risk-associated mutations in Alzheimer’s disease, Frontotemporal Dementia, Amyotrophic Lateral Sclerosis, Parkinson’s disease, Huntington’s disease and others have given the field a unique mechanistic insight into protein clearance processes in neurons. Through their broad implication in neurodegenerative diseases, molecules involved in these genetic pathways, in particular those involved in autophagy, are emerging as appealing therapeutic targets for intervention in neurodegeneration.

## Introduction

Neurodegenerative diseases are defined by the progressive and irreversible destruction of neurons, with age-associated cell death occurring through heterogeneous, only partially defined mechanisms. A varied range of behavioural, cognitive and physiological symptoms are associated with neurodegenerative diseases, dependent on the affected neuronal populations. The most common neurodegenerative diseases broadly cause two primary symptoms, cognitive decline such as the profound dementia presented in Alzheimer’s disease (AD), and motor system dysfunction such as the slowing of movement and eventual paralysis seen in Parkinson’s disease (PD). Almost without exception, effective preventative therapeutics are unavailable for neurodegenerative diseases, with only palliative treatments currently in use. As with most neurological disorders [1] neurodegenerative diseases are distributed globally with an increasing incidence correlating with the ageing populations, and associated with a growing health and socioeconomic burden.

A paucity of effective treatments for neurodegenerative diseases has led to an urgent search for candidate cellular mechanisms for therapeutic intervention. Protein turnover has long been implicated in many of the most common neurodegenerative diseases, through the discovery that several proteins genetically linked to familial forms of disorders form stable aggregates within cells. Well-described examples include AD associated amyloid plaques and hyperphosphorylated Tau containing neurofibrillary tangles, PD associated Lewy bodies and neurites, and cytosolic inclusions of Amyotrophic Lateral Sclerosis (ALS). The accumulation of these mono- and oligomeric peptides suggests ineffective cellular clearance of macromolecules, in particular via the endo-lysosomal and autophagic machinery. Emerging genetic and molecular biological evidence now suggests that both systems may be dysfunctional across a broad spectrum of neurodegenerative disorders, their contribution expanding beyond just the turnover of aggregation prone proteins in neurons (Table 1). Here we summarily review the evidence for a role of endo-lysosomal and autophagy dysfunction in progressive neurodegenerative disorders, using specific examples of their contribution from common disorders to illustrate key concepts.

**Table 1:** Neurodegenerative disease associated gene associated with autophagy and endo-lysosomal processes

Gene	Process	Evidence	Key References
Alzheimer’s Disease			
*APOE*	Early endosomes	GA	[2]
*BECN1*	Macroautophagy	BM	[3–5]
*BIN1*	Early endosomes	GA	[6]
*CD2AP*	Early endosomes	GA	[7]
*CLU*	Early endosomes	GA	[8, 9]
*EPHA1*	Early endosomes	GA	[10, 11]
*PICALM*	Early endosomes	GA	[8]
*PSEN1*	Recycling endosomes / Lysosomes	GA	[12]
*RAB7A*	Mitophagy / Late endosomes	BM	[13]
*SORCS1*	Retromer	GA	[14]
*SORL1*	Early endosomes / Retromer	GA	[14]
Parkinson’s Disease			
*DNAJC6*	Early endosomes / Retromer	GA	[15, 16]
*FBXO7*	Mitophagy	CM	[17, 18]
*GAK*	Late endosomes	GA	[19]
*GBA*	Mitophagy, Lysosome	GA	[20]
*LRRK2*	Mitophagy / Macroautophagy / Chaperone-mediated mitophagy (CMA)	CM	[21–23]
*PINK1*	Mitophagy	CM	[24, 25]
*PRKN*	Mitophagy	CM	[26, 27]
*RAB29/RAB7L1*	Late endosomes	GA	[28]
*SNCA*	Macroautophagy	CM/BM	[29]
*SYNJ*	Early Endosomes	GA	[30, 31]
*UCH-L1*	CMA	GA/BM	[32, 33]
*VPS35*	Retromer	CM	[34, 35]
Huntington’s Disease			
*ATG7*	Macroautophagy	GA	[36, 37]
*BECN1*	Macroautophagy	BM	[38]
*HTT*	Early endosomes / Recycling endosomes	CM / BM	[39, 40]
Amytrophic Lateral Sclerosis / Frontotemporal Dementia			
CHMP2B	Early Endosomes	GA / CM	[41, 42]
*OPTN*	Macroautophagy / Mitophagy	GA	[43]
*p62/SQSTM1*	Macroautophagy	CM	[44, 45]
*TBK1*	Macroautophagy / Mitophagy	CM	[46, 47]
Charcot Marie Tooth			
*RAB7A*	Mitophagy / Late endosomes	CM	[48]
*SH3TC2*	Recycling endosomes	CM	[49]
Niemann-Pick Disease			
*NPC1*	Lysosomes	CM	[50, 51]
*NPC2*	Lysosomes	CM	[52, 53]

*BM* Biomarker: genes with histological, molecular or biochemical evidence for contribution of gene in neurodegenerative disease; *CM* Causative Mutation: Genes associated with hereditary forms of neurodegenerative disease; *GA* Genetic Association: Genes where association with neurodegenerative disease has been made through -*omics* research

## Autophagy

Autophagy is a process of ‘self-eating’ through which unwanted or toxic macromolecules and organelles are sequestered and delivered to the lysosome to generate raw materials including proteins, lipids, carbohydrates and nucleic acids for use in metabolic processes. In most cell types, autophagy functions primarily in response to starvation [54] and some forms of apoptosis [55]. However, in post-mitotic neurons, where the programmed death and replacement of unhealthy cells is not a viable option, autophagy takes on a more crucial role in maintaining normal cellular homeostasis, in particular the critical turnover of misfolded proteins and damaged organelles. This is demonstrated by observations of increased autophagy in response to acute brain damage such as strokes and traumatic brain injuries, however there is still controversy as to whether this response is homeostatic or pathological (reviewed by [56]). There are three mechanistically distinct forms of autophagy that function within neurons; macroautophagy, chaperone-mediated autophagy (CMA) and microautophagy, each of which have been implicated in maintaining normal neuronal function or in neurodegeneration.

### Macroautophagy signalling cascade

**Fig. 1:**
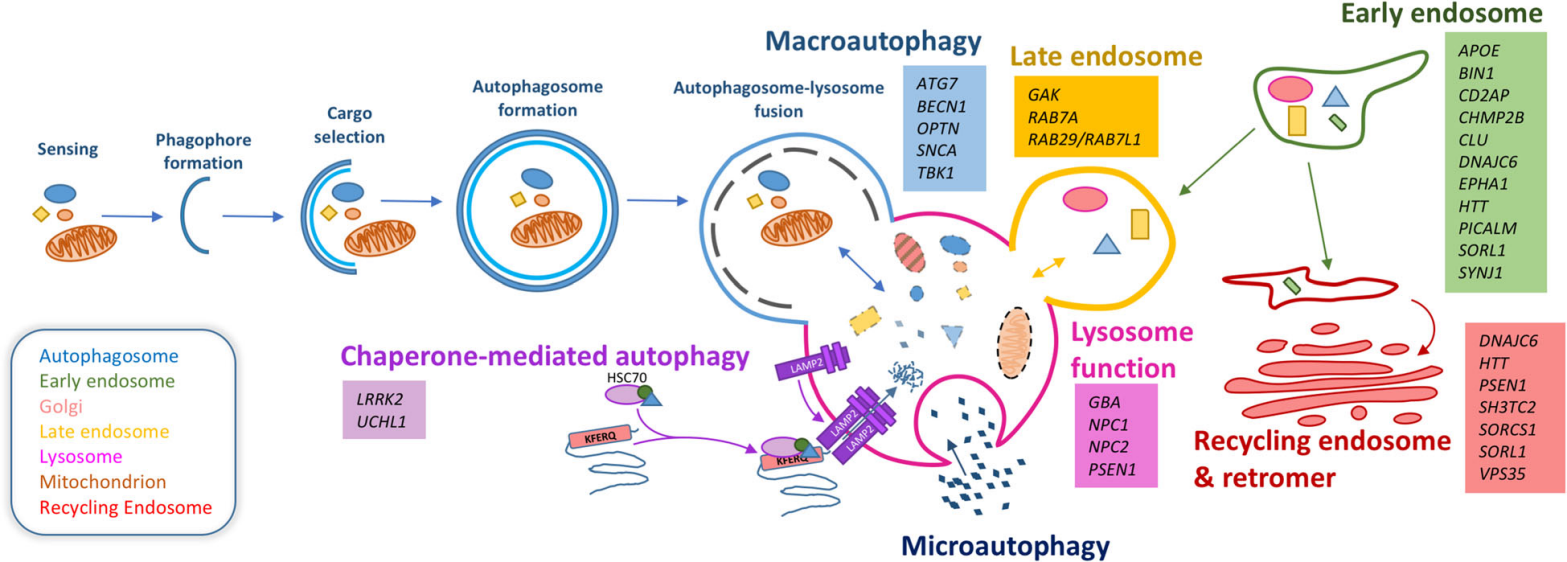
Autophagy and endo-lysosomal mechanisms and related genes associated with neurodegenerative diseases. Macroautophagy begins with formation of an isolation membrane to engulf cargos selected for degradation. Elongation of the isolation membrane results in formation of the double membrane autophagosome marking the final step before lysosomal fusion and degradation. In parallel the endosomal system sorts molecules for either recycling or targeting to the lysosome, with chaperone-mediated autophagy (CMA) and microautophagy also delivering cargos to the lysosomes. Hydrolytic enzymes within the acidic lysosomal lumen digest the target and the constituents resulted from this are released into the cellular cytoplasm. Neurodegenerative disease causing or associated genes affecting various stages of autophagy are listed alongside the process in which they are involved. For additional information relating to disease association of listed genes, refer to Table 1

During macroautophagy, macromolecules and organelles such as mitochondria and peroxisomes are sequestered within specialised vesicles and digested for removal or generation of raw material (Fig. 1). Macroautophagy is a complex sequential process composed of multiple steps which are generally considered consistent between cell types, predominantly facilitated by a cascade of Autophagy Related Genes (ATG). Sensing is the crucial first step in autophagy induction, where the cell makes a choice to induce degradation of toxic or superfluous cellular components. In normal healthy physiological conditions, the serine-threonine kinase mammalian target of Rapamycin (mTOR), the master sensor for autophagy, forms the mTOR complex (MTORC1) to promote cell growth [57]. Depleted levels of cellular cyclic adenosine monophosphate (cAMP) activate 5′-adenosine monophosphate activated protein kinase (AMPK), which in turn phosphorylates unc-51 like autophagy activating kinase 1 (ULK1), promoting it to form a complex with Focal Adhesion Kinase Family Kinase-Interacting protein Of 200 KDa (FIP200), ATG13 and ATG101 [58, 59]. Initiation/nucleation triggers formation of the ‘phagophore’, a lipid double membrane produced to encapsulate the target cargo, restricting it to a smaller cytoplasmic region for further processing. To enable phagophore formation, ULK1 phosphorylates and activates the vacuolar protein sorting 34 (VPS34) complex, consisting of the class III phosphatidylinositol-3-kinase VPS34, Beclin1, VPS14 and VPS15 [60]. The activated VPS34 complex enriches the isolation membrane with phosphatidylinositol 3-phosphate (PI(3)P), recruiting additional autophagy machinery. The phagophore next undergoes elongation, facilitated by two processes. Firstly, phosphatidylethanolamine is covalently bound to cytosolic Microtubule Associated Protein 1 Light Chain 3 and GABARAP family proteins (herein LC3-I), producing an autophagosome-associated LC3-II [61]. Secondly a complex of ATG5-ATG12-ATG16 associates with the isolation membrane, allowing it to entirely enclose the whole target organelle [62]. Selection of cargos occurs in parallel to sensing, initiation and elongation, marking substrates for autophagy. Proteins are targeted for autophagy by ubiquitination and labelling primarily with p62/Sequestosome-1 (p62), which through an ATG8 interaction motif/LC3 interacting region (AIM/LIR) [63, 64] recruits LC3-II to the isolation membrane [65]. Other cargo recognition proteins including Neighbour Of BRCA1 Gene 1 (NBR1), Nuclear Domain 10 Protein 52/ Calcium Binding And Coiled-Coil Domain 2 (NDP52), and Optineurin (OPTN), also contribute to specific targets for autophagy [66]. Once target cargos are bound by LC3-II, further initiation machinery is recruited. Closure of the membrane leads to formation of a double membraned vesicle called an ‘autophagosome’, containing the target cargos. Since their formation can occur in synapses and neurites significant distances from the neuronal soma [67], transport of autophagosomes is often necessary for their delivery to appropriate cellular compartments for degradation. Autophagosomes finally undergo fusion with late-endosomes or lysosomes to deliver substrates for hydrolytic enzymatic degradation.

### Macroautophagy

Macroautophagy is a highly conserved process [68, 69] and unsurprisingly several key molecules and mechanisms are associated with neuronal dysfunction and degenerative conditions. The critical importance of autophagy in neuronal health is best documented in model organisms deficient for genes required for the initial steps of autophagy. The ULK1 homologue, serine/threonine-protein kinase unc-51 was first identified in a *C. elegans* screen for genes associated with ‘uncoordinated’ phenotypes, with its dysfunction resulting in incomplete developmental axon outgrowth and elongation [70], clearly demonstrating the importance of autophagy in normal neurodevelopment. Current research however, also suggests that aberrant autophagy plays a fundamental role in ageing and neurodegeneration. Conditional deletion of essential autophagy genes in mice has demonstrated the critical requirement for neuronal autophagy in adult animals. Mice lacking neuronal expression of the autophagosome membrane elongation genes ATG5 [71] or ATG7 [72] are viable into adulthood, however they show significantly reduced autophagy, associated with progressive motor dysfunction and neurodegeneration. A notable observation from both ATG5 and ATG7 deletion models was the formation of intraneuronal inclusion bodies composed of ubiquitinated protein. Large protein aggregates are considered a hallmark histopathological feature of many neurodegenerative disorders, though their precise contribution (or even protection) is poorly defined.

Genetic evidence from several neurodegenerative diseases supports a contribution for protein accumulation in pathogenesis, with hereditary mutations in many aggregation prone proteins, and also dysfunction of cargo selection genes required for targeting inclusions to the autophagy system. Natively unfolded alpha-synuclein [29] forms Lewy bodies and neurites in PD and associated Parkinsonisms [73], with hereditary point mutations increasing aggregation propensity [74]. Hyperphosphorylation of Microtubule Associated Protein Tau (Tau) accumulates in neurofibrillary tangles [75–77] in several neurodegenerative diseases including AD and familial forms of Frontotemporal Dementia (FTD) [78]. Extracellular amyloid beta (Aβ) plaques are a hallmark of AD, composed of fragments of amyloid precursor protein (APP) [79, 80] generated by presenilin secretases (PSEN1/2) [81], with *APP* [82, 83] and *PSEN* genes [84, 85] mutated in familial AD. Both familial and sporadic Amyotrophic Lateral Sclerosis (ALS) is associated with cytosolic aggregation of proteins, most frequently TDP-43 [86, 87], with FUS [88] and SOD1 [89–91] seen in some familial cases. Mutations resulting in repeat expansion are also associated with protein aggregation such as the ALS/FTD linked gene *C9ORF72* [92–94], or polyglutamine (polyQ) expansions seen in several disorders, most prominently Huntingtin (HTT) in Huntington’s Disease (HD) [39, 95]. Typically intracellular inclusion bodies are ubiquitinated and labelled with autophagy receptor proteins, most commonly p62 [96], suggesting autophagy plays an active role in their clearance. Mutations in *p62* [44, 45] and *OPTN* [43] have been identified in ALS and FTD, directly implicating effective protein targeting for clearance in disease pathogenesis.

Mutations in core autophagy genes have not been identified as directly causative in any common neurodegenerative disorders, though some rare conditions have been reported [97]. There is however a wealth of data implicating the mis-regulation of autophagy sensing and initiation/nucleation in neurodegenerative disorders, particularly HD. Experiments in cellular [98], *Drosophila* and mouse models of polyQ expanded HTT [99] have demonstrated that promoting autophagy through pharmacological inhibition of mTOR is sufficient to rescue phenotypes associated with HD toxicity. Contrasting evidence suggests that expression of the mTOR activators RAS homolog enriched in brain (Rheb) or RAS homolog enriched in the striatum (Rhes) can also alleviate symptoms in HD mouse models [100], while only transient protection is seen in R6/2 mouse treated with mTOR inhibitors [101]. The contribution of autophagy to HD is complicated by the fact that mTOR is found within poly-Q rich protein aggregates [99] and that polyQ-expanded HTT enhances mTORC1 activity [102], suggesting a direct interaction of mTOR with HTT. PolyQ-expanded HTT can disrupt nucleation of the isolation membrane, by impairing the phosphorylation of Beclin-1 associated ATG14 and VPS34 complex activity [103]. Pro-nucleation has also been implicated in polyQ-repeat associated Machado-Joseph disease/spinocerebellar ataxia type 3, with increased expression of the VPS34 complex component Beclin-1 showing protective activity in mouse models [104, 105]. Beclin-1 mediated nucleation may also contribute to the pathology of AD. In transgenic mouse AD models expressing human APP, reduction of Beclin-1 expression leads to increased intraneuronal Aβ accumulation, extracellular Aβ deposition and neurodegeneration [3]. Intriguingly, peptide fragments produced through caspase cleavage of Beclin-1 have been detected in the brains of AD patients and murine models, which exacerbated neurodegenerative phenotypes when overexpressed [4]. Conversely, caspase-resistant Beclin-1 was found to be neuroprotective, suggesting some post-translationally processed species of Beclin-1 may themselves be toxic [4].

A direct function of ALS/FTD gene *C9ORF72* in the initiation of autophagy has also been suggested recently. ALS/FTD can manifest through a 5′ hexanucleotide repeat expansion in the *C9ORF72* gene, which can contain several thousand repeats generating both RNAi and protein products that accumulate over time [92, 94]. In addition to clear gain-of-function pathologies, mutant *C9ORF72* alleles may also reduce expression of some isoforms of the gene, suggesting partial loss-of-function may partially contribute to disease [92]. C9ORF72 has been found to interact with RAB1A, a RAB GTPase effector molecule required for the recruitment of ULK1 complex to the phagophore [106]. Decreased levels of autophagy have been reported in neurons derived from C9ALS/FTD patients, and reduction of C9ORF72 expression in cultured neurons was found to attenuate autophagy and accumulation of intracellular p62 puncta, indicative of protein accumulation [106, 107].

### Chaperone-mediated autophagy

Chaperone-mediated autophagy (CMA) is a selective form of autophagy, whereby peptides carrying a KFERQ-like motif are recognised by cytoplasmic chaperone proteins, which then deliver the target directly to lysosomes for degradation [108]. Target peptides are bound by cytoplasmic chaperones including Heat shock protein 90 (HSP90), delivered to the lysosome-associated membrane protein 2A (LAMP2A) receptor on the lysosomal membrane and transported into the lysosome lumen for hydrolytic degradation. Unlike micro- and macroautophagy, CMA is not evolutionarily conserved and has only been observed in mammalian cells [109, 110].

CMA contributes to the clearance of proteins associated with several neurodegenerative disorders [111, 112], with compelling evidence to suggest a role in the dopaminergic neuron loss seen in PD. Several genes genetically associated with familial forms of PD appear to disrupt CMA. The natively unfolded alpha-synuclein peptide is a substrate for CMA [113], however both stabilised dopamine-bound peptides, [114] and PD-associated mutant species [113] are ineffectively degraded through this process. Ubiquitin carboxyl-terminal esterase L1 (UCHL-1), has been shown to interact with heat shock protein 70 (HSC70), HSP90 and LAMP2A, with disease associated mutations further increasing binding and impeding CMA of alpha-synuclein [115]. PD-associated Leucine Rich Repeat Kinase 2 (LRRK2) also appears to be degraded through CMA, with the PD associated mutations rendering the protein a poor substrate but also impeding the CMA translocation complex [116]. Most recently, PD-associated deglycase DJ-1, which functions in neuronal response to oxidative stress and mitochondrial turnover, has also been found to undergo CMA-mediated degradation, with a preference for non-functional oxidised forms [117]. Reduced CMA and turnover of non-functional DJ-1 was associated with increased mitochondrial dysfunction and cell death in repose to toxin induced oxidative stress [117]. CMA and PD have also been associated through the degradation of myocyte enhancer factor 2D (MEF2D), a transcription factor that contributes to neuronal survival under stress [118]. Inhibition of CMA through knockdown of HSC70 or LAMP2A results in accumulation of cytoplasmic non-functional MEF2D in neuronal cultures, with increased cytoplasmic MEF2D also reported in alpha-synuclein transgenic mice and PD patient tissues [119]. Taken together, these findings suggest processing of PD-associated peptides through CMA may be a contributing factor in disease pathogenesis and progression and that this process may be critical for the maintenance of dopamine neurons in particular.

### Microautophagy

Microautophagy is the least well characterised of the three forms of autophagy, with its role in neurodegeneration mostly unexplored. In this process, proteins entering the endo-lysosomal system through invagination are engulfed by the late endosome and lysosomal membrane [120]. The synapse appears to be a particularly vulnerable neuronal compartment in many neurodegenerative disorders, in part due to the constant turnover of SNARE proteins required for neurotransmitter release, which can form dysfunctional neurotoxic species [121]. Experiments in *Drosophila* have demonstrated that an endosomal form of microautophagy can be perturbed through knockdown of the synapse enriched chaperone HSC70–4, required for recognition of the peptide degradation motif, resulting in significantly perturbed neurotransmitter release [122]. As microautophagy appears to support normal neuronal function, particularly at sensitive synaptic terminals, further investigation should be conducted in the context of neurodegenerative disorders to define its contribution.

### Selective autophagy

Autophagy mechanisms can also be subclassified into those involving selective degradation of specific organelles, such as peroxisomes (pexophagy), nuclei (nucleophagy) and endoplasmic reticulum (ER-phagy), as well as those involving degradation of molecular materials such as lipids (lipophagy), stress granules (granulophagy) and myelin (myelinophagy) (reviewed by [123]). Autophagy receptors for selective targeting of organelles which, under specific conditions, link these organelles with the cellular autophagy machinery leading to their destruction, are being continuously discovered. Receptors important for pexophagy include NBR1 [124], Atg30 [125] and Atg36 [126], whereas FAM134B [127] and Atg40 [128] are required for ER-phagy. Once bound to these adaptors, cargos enter the autophagy cascade for lysosomal degradation.

**Fig. 2:**
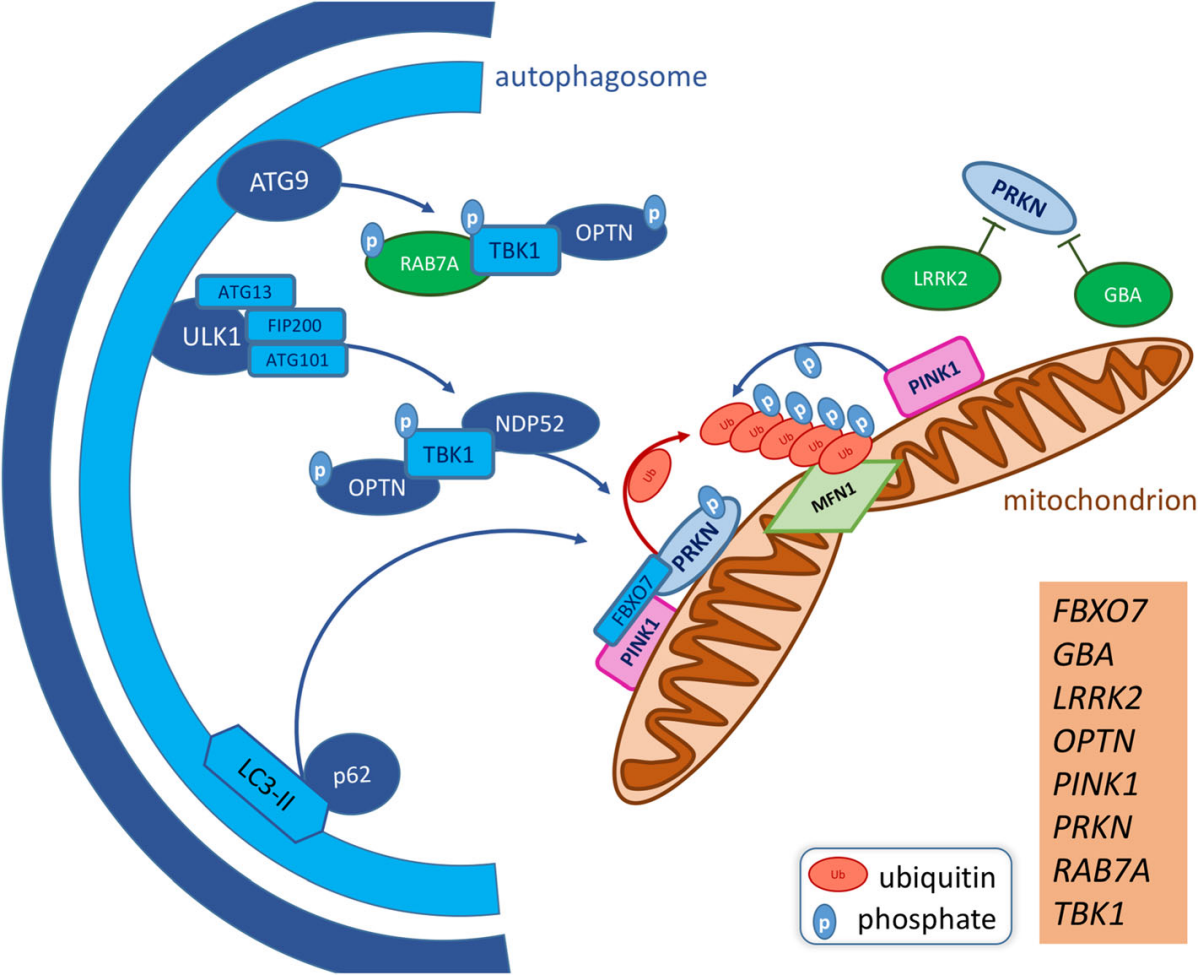
Mitophagy and related genes associated with neurodegenerative diseases. Dysfunctional mitochondria are targeted for autophagic clearance by a number of specific adapters which are associated with neurodegenerative disorders. Upon depolarisation, PTEN-induced kinase 1 (PINK1) accumulates on the mitochondrial outer membrane (MOM), where it phosphorylates Ser65 of ubiquitin and the ubiquitin-like domain of PRKN. pS65-Ub acts as a positive-feedback mechanism for the further recruitment of PRKN to the MOM and activation of its E3 ubiquitin-ligase activity. PRKN ubiquitinates a number of targets on the MOM, including mitochondrial fusion proteins such as Mitofusin1 (MFN1), decorating the damaged organelle in poly-ubiquitin chains. F-Box Only Protein 7 (FBXO7) also participates in MFN1 ubiquitination. PINK1, PRKN and pS65-Ub chains on the MOM facilitates the recruitment of autophagy adapters Phosphotyrosine-Independent Ligand For The Lck SH2 Domain Of 62 KDa (p62), Nuclear Domain 10 Protein 52 (NDP52) and Optinuerin (OPTN). Parkinson’s disease-associated mutations in β-glucocerebrosidase (GBA) and Leucine Rich Repeat Kinase 2 (LRRK2) are considered to impair PRKN-mediated mitophagy. Phosphorylation of ALS-associated TBK1 in response to mitochondrial damage is dependent on NDP52 and OPTN recruitment, but subsequently increases the affinity of OPTN for poly-ubiquitin on the MOM. TBK1 also phosphorylates RAB7A, which in turn facilitates the recruitment / formation of ATG9 vesicles. The ULK1 complex and ATG9 vesicles are recruited / form de novo at damaged mitochondria and initiate autophagic engulfment. This is enhanced by the recruitment of LC3-II by p62. Neurodegenerative disease causing or associated genes affecting various stages of mitophagy are listed. For additional information relating to disease association of listed genes, refer to Table 1

Whilst the contribution of most cargo selective forms of autophagy to neuronal health is largely unexplored, mitophagy, perhaps the most thoroughly characterised, has been strongly implicated in neurodegenerative disease. Mitophagy is the process by which dysfunctional mitochondria are selectively targeted by autophagosomes and degraded via autophagosome-lysosome fusion, facilitating a quality-control mechanism which maintains a healthy mitochondrial network (Fig. 2). Due to their high metabolic demand and post-mitotic state, neurons are particularly sensitive to mitochondrial dysfunction and thus mitophagy is vitally important in this cell type. Like other forms of selective autophagy, the targeting of mitochondria for mitophagy occurs though a mechanism which parallels that of general macroautophagy cargo targeting, but with specific adapters that allow for the selective targeting of damaged organelles. The canonical mitophagy model is that mitochondrial insult results in the dissipation of mitochondrial membrane potential (ΔΨm), followed by a block of PTEN-induced kinase 1 (PINK1) import into the intermembrane space, where it is usually cleaved by Presenilin Associated Rhomboid-Like (PARL) [129]. PINK1 accumulates on the mitochondrial outer membrane (MOM) and phosphorylates ubiquitin at Ser65 (pS65-Ub), leading to the recruitment of Parkin E3 Ubiquitin Protein Ligase (PRKN) from the cytosol [130]. PINK1 also phosphorylates PRKN at Ser65 of its ubiquitin-like domain, stimulating PRKN E3 ubiquitin ligase activity [131]. This triggers a positive-feedback mechanism during which subsequent PRKN recruitment and ubiquitination of MOM proteins [132, 133] results in the recruitment of AIM/LIR autophagy adapters including p62, OPTN and TAX1 Binding Protein 1 **(**TAX1BP1). The kinase domain of PINK1 has been shown to recruit OPTN and NDP52 independent of PRKN and recruitment of these two adapters is essential for mitophagy [134]. Though responsible for recruiting LC3-II to the poly-ubiquitinated MOM [135], p62 is dispensable [134, 136] but can improve the efficiency of mitochondrial incorporation into autophagosomes at a later stage in the process. The ULK1 complex transiently assembles at depolarised mitochondria [137], in a PRKN-dependant, LC3-II-independent fashion. ATG9A vesicles are also recruited / formed de novo at depolarised mitochondria, independently of ULK1 recruitment. ULK1 and ATG9A foci only partially co-localise at mitochondria and neither are required for the recruitment of LC3-II, though both are required for mitophagy to occur [137].

The most well-established association between defective mitophagy and neurodegeneration is with PD since *PRKN* was discovered as the causation of autosomal-recessive juvenile parkinsonism (ARJP) in a Japanese population [26, 27] and *PINK1* was subsequently identified as a second ARJP associated gene [24, 25]. Initial functional characterisation of both genes was performed in *Drosophila,* demonstrating loss of function mutations in the *Drosophila PRKN* homologue *parkin* cause aberrant mitochondrial morphology in energy demanding cell types, such as sperm, flight muscle and, more relevant to PD, dopaminergic neurons [138, 139]. Similar phenotypes were observed in *Pink1* mutant *Drosophila* and genetic epistasis experiments showed that overexpression of *parkin* rescued *Pink1* mutant phenotypes but not vice versa, placing PRKN downstream of PINK1 in a common pathway of mitochondrial quality control [140–142]. *PINK1* and *PRKN* patients feature the loss of DA neurons of the substantia nigra pars compacta and mitochondria are enlarged in induced pluripotent stem cells (iPSC)-derived DA neurons from these patients [143]. Taken together, PINK1 and PRKN genetic and experimental evidence strongly associate loss of normal mitophagy with ARJP.

Histopathological post-mortem analysis of PD patient brains also suggests disrupted turnover of mitochondria. Mitochondrial complex I defects in the post-mortem substantia nigra are a hallmark of PD pathology [144], indicating that deficient mitochondrial quality control is a common feature across familial and sporadic cases of PD (reviewed [145]). A signature of damaged mitochondria, polymeric pS65-Ub, accumulates in cytoplasmic granules, beaded neurites and granulovacuolar degeneration bodies with age in healthy individuals [146, 147]. In sporadic PD and Dementia with Lewy Bodies patients, these structures have been identified in the proximity of Lewy bodies. Their abundance positively correlates with both age and Braak stage, demonstrating age and disease-associated increases in mitochondrial quality control [147]. Expectedly, given that PINK1 and PRKN are responsible for generating pS65-Ub chains on the MOM, pS65-Ub positive structures are markedly reduced in *PINK1* and *PRKN* post-mortem brains, inferring that defects in mitophagy are observed in these patients [146, 147].

Mitophagy-related roles can further be attributed to several other PD-associated genes. Mutations in another E3 ubiquitin ligase, F-Box Only Protein 7 (FBXO7), were identified as the cause of parkinsonian pyramidal syndrome, a rare form of ARJP which presents with pyramidal tract dysfunction [17, 18]. FBXO7 enhances PRKN recruitment to depolarised mitochondria and also participates in the ubiquitination of Mitofusin 1 (MFN1), facilitating the segregation of damaged mitochondria from the healthy mitochondrial network [148]. The most frequent cause of autosomal dominant PD is the G2019S hypermorphic variant of *LRRK2*. Cold-shock induced mitophagy is impaired in fibroblasts derived from patients with either *PRKN* mutations or the G2019S *LRRK2* variant. This effect is reversed by treatment with LRRK2 inhibitor LRRK2-in-1 in G2019S *LRRK2* but not *PRKN* loss-of-function fibroblasts [149]. LRRK2-in-1 may also protect against oxidative stress by restoring basal mitophagy levels in a subset of sporadic PD derived fibroblasts [150].

Mitophagy has more recently been implicated in other neurodegenerative diseases. *PINK1* has been identified in GWAS of genetic modifiers of HD progression, along with a number of regulators of mitochondrial fission/fusion dynamics [151]. In a *Drosophila* model of HD*, Pink1* overexpression rescues mitochondrial morphology, conveys neuroprotection and extends lifespan, indicating that defects in mitophagy may also contribute to aspects of HD pathogenesis [152]. ALS-associated mutations in OPTN or TANK-binding protein 1 (TBK1) block efficient clearance of depolarised mitochondria in PRKN-expressing HeLa cells, indicating that both of these proteins are important, though not essential, in mitophagy [153]. TBK1 is rapidly phosphorylated and activated upon mitochondrial damage and this is dependent on the mitochondrial recruitment of NDP52 and the ubiquitin binding domain of OPTN. In turn, p-TBK1 phosphorylates OPTN, enhancing its affinity for polyubiquitin and thus its retention at depolarised mitochondria [154]. p-TBK1 also phosphorylates RAB7A [155] required for ATG9-vesicle formation and efficient mitophagy in PRKN-expressing HeLa cells [155–157]. Some ALS-associated missense mutations in TBK1 abolish its phosphorylation, activation and ability to phosphorylate OPTN [158], thus defects in mitophagy could play a role in the pathogenesis of patients with these mutations.

Changes in mitophagy may also occur in diseases where genetic evidence does not clearly suggest a mitochondrial contribution to pathology. In AD, post-mortem hippocampal tissues exhibit strikingly lower levels of mitophagy, assessed by mitochondria-lysosome co-localisation and visualisation of mitophagy events by transmission electron microscopy. These deficits correlate with decreased PINK1, p-TBK1 and p-ULK1 in the same samples and also in iPSCs derived from *apolipoprotein E4* (*APOE4)* and *APP*-mutation carrying patients [159]. Furthermore, there is evidence that upregulation of mitophagy may protect against AD phenotypes. Urolithin A (UA), a metabolite produced in the gut from the ellagitannin class of polyphenols found in pomegranate, raspberries and walnuts, upregulated mitophagy in nematodes and rodents in a manner dependant on PINK1/PRKN and independent of general macroautophagy [159, 160]. UA treatment ameliorated learning and memory defects in both Aß and hyperphosphorylated Tau in a *C. elegans* models, and improved cognition in mouse models of AD [159]. Intriguingly this study found microglial activation and neuroinflammation were reduced upon UA treatment in APP-PSEN1 mice, suggesting mitophagy deficits are tied to chronic inflammation in the brain, a hallmark of many neurodegenerative diseases. The anti-inflammatory cytokine Interleukin 10 was increased in hippocampal microglia of these mice upon UA treatment, in a PINK1-dependent fashion, indicating that this anti-inflammatory response is also likely dependent on mitophagy. Interleukin 10 has previously been shown to promote mitophagy through inhibition of the mTOR pathway in lipopolysaccharide-activated macrophages, maintaining a healthy mitochondrial network and a metabolic profile based on oxidative phosphorylation as opposed to glycolysis [161]. Microglial activation is associated with a respiratory switch from oxidative phosphorylation to glycolysis, facilitated by the glucose transporter GLUT1. The GLUT1-specific inhibitor STF31 supresses neuroinflammation and neurodegeneration in a mouse model of light-induced retinal degeneration [162]. These studies thus identify a promising strategy for combatting both mitochondrial dysfunction and chronic neuroinflammation in neurodegenerative disease, through upregulation of mitophagy and rebalancing of metabolic state.

### Therapeutic targeting of autophagy in neurodegeneration

Through their implication in a broad range of neurodegenerative disorders, endo-lysosomal and autophagy mechanisms have become appealing targets for therapeutic intervention [163]. Autophagy targeting compounds fall in two broad categories, acting through mTOR-dependent or -independent mechanisms. Modulation of mTOR-dependent autophagy via inhibition of mTORC1 with rapamycin has been widely explored across a spectrum of human diseases, including various forms of cancers, auto-immune and neurodegenerative disorders [164]. Rapamycin possesses strong immunosuppressant and anti-proliferation properties which, though beneficial for treatment of cancer and autoimmune disorders, are undesirable for chronic treatment of neurodegenerative disorders. As rapamycin has been found beneficial in treatment of neurodegeneration in preclinical models [165], attempts to circumvent its immunosuppressant activity have been made through “Rapalog” derivative molecules, several of which have been demonstrated to improve phenotypes in models of neurodegenerative disorders including HD [99], spinocerebellar ataxia type-3 [166] and FTD-associated tauopathy [167].

Several mTOR-independent modifiers of autophagy are gaining interest as therapeutics, with AMPK activating molecules such as trehalose and metformin proving effective in reducing neurodegenerative phenotypes in models of AD [168, 169], ALS [170–172], HD [173, 174] and tauopathies [175]. Cellular targets not directly associated with the core autophagy machinery have also been found to modify neurodegeneration, including Estrogen Related Receptor α [176] and cAMP [177, 178]. Interestingly, the widely used AD-therapeutic memantine has emerged from a screen of clinically approved molecules which enhance autophagy [179], suggesting a potential mode of action for the drug which may be repurposed in other neurodegenerative disorders.

## Endosomes

Endosomes capture surface molecules through internalisation of the plasma membrane, or acquire cargo intracellularly following *trans*-golgi trafficking. Multiple checkpoints along the endosomal pathway either designate cargos for degradation at the lysosome or recycle them back to the plasma membrane or golgi via the retromer complex [180, 181]. Endosomes exist in three specific states: early (also called sorting), recycling, or late depending on their post internalisation stage and association with distinct Rab guanosine triphophatases (RAB GTPases) [182].

### Early endosome

The early endosome (EE) serves as the primary sorting compartment of the endocytic pathway, receiving extracellular material, lipid membranes and membrane-bound proteins from small endocytic vesicles, formed from specialised clathrin-coated invaginations of the plasma membrane. Upon their delivery to the EE, cargos are separated within minutes and assigned for either degradation or recycling. Proteins destined for recycling back to the plasma membrane first cluster within tubular EE extension membranes, whereas the larger and rounder EE compartment houses proteins targeted for degradation. Retrograde transport of cargos from the EE to the trans-golgi network is facilitated by the Retromer complex, which consists of VPS26-VPS29-VPS36 cargo recognition and sorting nexin (SNX) membrane recognition components [183]. Endosomal cargo separation is regulated primarily by RAB4 [184] and RAB5 [185], in addition to some other less well characterised GTPases including RAB10 [186], RAB14 [187], RAB21 [188] and RAB22 [189]. These RAB proteins facilitate either the recruitment of additional RABs to enable vesicle maturation or provide a platform for other proteins and protein complexes to dissociate and re-associate with the vesicle membrane [190] for trafficking or sorting purposes. The PI(3)P rich EE membrane itself is also generated through recruitment of PI 3-kinase VPS34 by RAB5 [191].

Initial endocytosis is disturbed in several age-dependent neurological disorders, notably PD where mutations have been identified in several EE genes. The synaptic enriched inositol-phosphatase Synaptojanin 1 (SYNJ1) binds clathrin and associated proteins, likely contributing to the uncoating of clathrin coated vesicles. Loss of SYNJ1 is associated with dysfunctional endocytosis [192, 193], through disruption of the earliest stages of EE formation [30, 31]. Indeed, enlarged EEs and altered trafficking have been seen in fibroblasts derived from early onset PD patients carrying SYNJ mutations [194]. Endocytosis in PD may further be perturbed by disruption of DnaJ/Heat Shock Protein Family (HSP40) co-chaperone (DNAJC) proteins [195], notably DNAJC6/Auxilin-1 and DNAJC13/RME-8. Neuron-specific DNAJC6/Auxilin-1 interacts with HSC70, facilitating the uncoating of clathrin vesicles [15, 196], whilst DNAJC13/RME-8 decreases retromer-mediated cargo transport sorting through interaction with SNX1 thereby preventing the formation of the necessary tubular structure of the EE membrane [197, 198]. Disruption of retromer activity has also been directly implicated in PD through mutations in the retromer complex gene VPS35 [34, 35]. PD-associated mutations in LRRK2 have also recently been found to alter expression of essential endocytic proteins and also impair endocytosis of clathrin-associated synaptic vesicles in patient derived dopaminergic neuron cultures [199]. Lipophilic and aggregation prone alpha-synuclein may itself inhibit retromer recycling of some membrane proteins through blocking VPS17 and SNX3 from EE association [200].

Beyond PD, several other neurodegenerative disorders have been linked to the EE system. RAB5 interacts with Early Endosome Antigen 1 (EEA1), a soluble N-ethylmaleimide-sensitive fusion protein attachment protein receptors (SNARE) complex interacting protein, to enable vesicle fusion [191] and recruitment of HTT via HTT associated protein 40 (HAP40) to enable endosome motility [201]. The poly-Q repeat expansion found in disease associated alleles of the *HTT* gene has been found to upregulate HAP40, facilitating a shift of EEs from microtubules to actin thereby decreasing trafficking speeds [39, 40]. EE dysfunction may also contribute to juvenile-onset ALS through mutations to Alsin Rho Guanine Nucleotide Exchange Factor (*ALS2*) [202, 203]. *ALS2* contains several domains required for guanine-nucleotide exchange required for RAB activation. Loss-of-function mutations in the *ALS2* gene have been found to interfere with GDP/GTP exchange required by RAB5 [204], resulting in EE accumulation and trafficking abnormalities [205]. EE function has long been of interest in AD pathogenesis, initially due to enlargement of RAB5 positive vesicles being one of the earliest pathological events seen in patient tissue [206]. This is not surprising given that the EE pathway is compromised at many levels from the endocytosis of secretases residing on the plasma membrane, intracellular trafficking of key enzymes through the internalisation of extracellular Aβ. More recently, emerging evidence suggests that genes associated with an increased risk for developing late-onset sporadic AD may converge on microglia [207], with several endocytic genes potentially contributing to pathology. Proteins encoded by AD risk genes, including *Bridging Integrator 1* (*BIN1*) [6], *CD2AP*, *EPHA1* [10, 11]*, PICALM* [8]*, Sortilin Related Receptor 1* (*SORL1*) [14], amongst others [7, 208, 209] may all interfere with EE function. SNPs in *BIN1* represent one of the most common AD risk associated mutations after *APOE*. RAS and RAB interactor 1 (RIN1), a BIN1 interacting protein, functions as guanine nucleotide exchange factor (GEF) for the RAB5 GTPase family. This interaction was found to promote *epidermal growth factor receptor (EGFR) downregulation* [210]*.* SNPs associated with *BIN1* are also likely to affect other critical RAB5 dependent processes, which require further investigation.

Retromer-mediated sorting from the EE further controls intracellular shuttling proteins relevant to AD including APP and Beta-Secretase 1 (BACE1), which are required to generate Aβ [211]. Knockout of retromer associated *Vps35* in a mouse model of AD enhanced levels of amyloidogenic Aβ species [212], suggesting that retromer signalling from the EE is a negative regulator of Aβ production. This theory is bolstered by genetic evidence linking SNPs and gene expression of *Sortilin Related VPS10 Domain Containing Receptor 1 (SORCS1*) [213], a membrane homologue of SORL1, to impaired retromer-associated sorting which may lead to APP processing deficits [214]. SNPs in *APOE* [215] and *Clusterin* may also accelerate extracellular Aβ-uptake and clearance [216, 217] decreasing endocytosis capacity later in disease. Given the role of EE dysfunction in a diverse range of neurological disorders, this pathway may represent a common mechanism of either disease manifestation or disease progression.

### Recycling endosome

Portions of neuronal plasma membrane and residing surface receptors that have been internalised and lost through EE formation are replenished by recycling endosomes (REs). At the ultrastructural level, REs have a tubular formation and form a non-continuous network [218], identified in tissues through association with RAB11 [219]. Lipids to be recycled are sorted away from those ubiquitylated receptors and ligands that are destined for degradation due to the REs acidic environment (pH ∼ 6.0) [220]. Endosomal recycling can be rapid, occurring within 2–3 min or can take around 10 mins from initial endocytosis. Different RAB subtypes appear to be required for either fast or slow kinetics, with RAB35 associated with fast moving vesicles and RAB11 slow [221–223], although why these different mechanisms exist and under what cellular conditions they occur is not well understood.

Although the RE compartment is relatively understudied compared to EE, several links to neurodegenerative diseases have been made. The activity of recycling endosome associated RAB11 is at least in part controlled through interaction with HTT. The removal of GDP from RAB11 is compromised by mutant polyQ expanded HTT in human cells, leading to deficits in RE size and receptor recycling [224], impacting dendritic spine complexity in rodent models and patients [225] and electrophysiology, lifespan and locomotion in *Drosophila* models of HD [225, 226]. RAB11 and its role in regulating recycling endosome activity has been implicated in disease beyond HD. Charcot-Marie-Tooth peripheral neuropathy type 4C associated SH3 domain and tetratricopeptide repeats 2 (SH3TC2) [49] is considered a RAB11 effector protein, localising to GTP-bound species [227]. In CMT4C, mislocalisation of SH3TC2 and lack of RE trafficking is considered a causative feature of disease progression [227]. Increasingly targeting RAB11 activity is now considered a keen therapeutic target for HD, with potential benefits for other neurodegenerative diseases.

Another important molecule bound to the EE/RE following secretion from the golgi is the gamma-secretase component PSEN1. Although enhanced amyloidogenic Aβ production is likely to play a role in disease manifestation, a recent report also suggests that it is the accumulation of β C-terminal fragments which cause RE dysfunction [228]. In this model, mutant forms of PSEN1 and APP decrease RAB11 dependent trafficking from the cell body to the axon [228]. Hence neurons have a decreased capacity to deliver lipoproteins, receptors and transporters back to the plasma membrane in vulnerable sub-compartments. Lysosome restricted PSEN2, which also cleaves APP, may play a more important role in normal cellular Aβ production, with more toxic species generated by the mislocalisation of mutant PSEN1 from the EE/RE to the lysosome [229].

Genetic evidence indicates that recycling of specific proteins confers neurodegenerative disease specificity. However, general RE disruption may contribute to neuronal demise indirectly. Several substrates of the RE pathway suggest why its disruption is so clearly detrimental to neuronal function. RAB11 vesicles have been found to carry important neurotrophic factors, such as BDNF [230] and critical synaptic receptors, such as AMPA [231]. Although loss of recycling capacity may not initially drive cell death, it may be key to understanding why synapses are preferentially lost early in disease.

### Late endosome

Late endosomes (LEs) / multi-vesicular bodies (MVBs) are generated through the maturation of EEs. Endosomal Sorting Complex Required for Transport (ESCRT) complexes 0-III and several VPS proteins are also recruited to ubiquitinated surface molecules on the cytosol facing endosomal membrane. ESCRT complexes facilitate the invagination of endosome membrane proteins and lipids, producing a MVB, an endosome containing smaller intraluminal vesicles [232]. During this maturation process RAB5 and RAB4 dissociate from the endocytic membrane and RAB7 and RAB9 are recruited. Genetic and cell biological evidence suggest that adequate RAB7 function to initiate clearance through LE-lysosomes fusions may be a critical factor in maintaining normal neuronal function [233]. RAB7 can also assist the recruitment of the retromer to late endosomes through interaction with VPS35 [234]. LEs/MVBs acidify to pH levels of 6.0–4.9 [235] in the final step of the endocytic pathway before intraluminal cargos are delivered to the lysosome for degradation (discussed below).

Dysfunction of LE activity in neurodegenerative appears mostly restricted to PD, spearheaded by genetic association. GWAS approaches have identified *LRRK2* mutations as common risk factors for the development of sporadic PD [21, 22], in addition to mutations in *Cyclin G Associated Kinase* (*GAK*) and the LE associate *RAB7L1* [19, 236]*.* Highlighting the importance of the LE pathway in the maintenance of the large highly arborised dopamine neurons, a protein complex of LRRK2, RAB7 and GAK was previous uncovered by an unbiased protein-protein interaction experiment [237]. Overexpression of these molecules promotes protein clearance from the trans-Golgi network, suggesting that trafficking from golgi to LE is compromised in PD. LRRK2 was also found to impede cargo trafficking by prohibiting budding of the LE membrane to form smaller vesicles, via decreased RAB7 activity [238], a process exacerbated by PD associated mutations. LRRK2 kinase inhibition via small molecules increases lysosome formation [150], suggesting that LE dysregulation directly impacts on clearance. Loss of LRRK2 or RAB7 also downregulates VSP35 [239] which may further perpetuate the dysregulation of the endosomal pathway upstream of the LE. The ESCRT-III complex has also been implicated in neurodegeneration through its function to concentrate endosomal cargos into LE intralumenal vesicles. ALS/FTD associated charged multi-vesicular body protein 2B (CHMP2B) mutations [41, 42] were found to cause severe lysosome pathologies [240] and metabolic disturbances in neurons [241]. This evidence shows that LE perturbations may therefore lead to downstream lysosome-mediated clearance complications or initiate dysfunction in earlier in the endocytic pathway.

## Lysosomes

Lysosomes are the terminal compartment through which macromolecules are degraded and recycled to generate nutrients. Lysosomes are generated through the maturation of LEs, achieved via delivery of hydrolytic enzymes from the golgi and also active acidification of the lumen via the lysosomal vATPase hydrogen pump. Once acidified to approximately pH 5, over 50 hydrolytic enzymes, including a broad range of glycosidases and proteases degrade the contents. The matured lysosome is able to fuse with and degrade the contents of other vesicular compartments including endosomes, autophagosomes, amphisomes (fused endosome-autophagosome) and phagosomes (phagocytosed material). A wide range of cellular macromolecules can be processed through the lysosome, including nucleic acids, proteins, carbohydrates and lipids. Several aspects of lysosomal biology including enzymatic dysfunction and positioning have been implicated in neurodegenerative disorders.

### Lysosome function

As lysosomal degradation is one of the primary mechanisms of cellular waste removal, it is unsurprising that genes facilitating this essential process have been linked to a broad range of diseases. Lysosomal Storage Disorders (LSD) are a family of hereditary conditions in which substrates of lysosomal degradation accumulate within the lumen, caused by mutations in a range of lysosome specific hydrolases, enzymatic regulators, membrane proteins and transporters. Many of the > 50 genes linked to LSDs cause juvenile neurodegenerative disorders, though pathologies of the liver, spleen and bones are also common (reviewed [242]). Several LSDs which feature neurodegeneration are associated with mutations in hydrolytic enzymes responsible for the processing of specific lipids, resulting in their build up within the lysosomal lumen. Examples include the Neuronal ceroid lipofuscinoses (NCLs), a family of 14 genetically distinct, autosomal recessive LSDs that present juvenile onset vision loss, seizures, cognitive decline and motor dysfunction, unified by the accumulation within neuronal lysosomes of auto-fluorescent lipofuscin, a heterogenous mixture of oxidised lipids, proteins and carbohydrates [243]. Neuroinflammation and neuronal death are seen in juvenile onset Sandhoff disease and heterogenous onset Tay-Sach’s disease, both GM2-gangliosidosis disorders caused by accumulation GM2-gangliosides within the lysosomal lumen [244].

The sphingolipidosis Gaucher’s disease is of particular interest due to implications in the pathogenesis of PD. Autosomal recessive Gaucher’s disease is characterised by the accumulation of glucosylceramide (GluCer) due to mutation of the β*-glucocerebrosidase* (*GBA*) gene [245], with progressive neurological dysfunction is seen in the severe early-onset type II and milder late-onset type III forms. In addition to accumulation of lysosomal GluCer, misfolded mutant GBA accumulates in the ER [246, 247], with mutant GBA associated with activated unfolded protein response in model systems [248–250]. There is a wealth of emerging data to suggest a strong association between impediment of lysosomal enzymatic function and synucleinopathies, in particular PD [251]. Genetic studies of PD patients have identified a strong association with heterozygosity for GBA loss-of-function mutations and increased risk of developing PD [252]. Both wild type alpha-synuclein and PD associated variants interact with lipids [253]. Dysfunction of GBA can disrupt alpha-synuclein function [254] and exacerbate its aggregation [255], with GluCer stabilising the peptide in oligomeric species and promoting its aggregation [256]. Alpha-synuclein is degraded through the lysosome via chaperone-mediate autophagy (see above) [257, 258]. Further still, accumulation of alpha-synuclein itself is able to inhibit lysosomal GBA function, suggesting a feed-forward loop of alpha-synuclein aggregation promoting lysosomal dysfunction and further accumulation of aggregated protein [256]. Intriguingly, PD-associated GBA variants GBA^L444P^ and GBA^N370S^ can also impede normal PRKN ubiquitination of mitochondrial substrates [259, 260], and heterozygous GBA^L444P^ mutations decrease the delivery of the mitochondria to lysosomes [261]. As mitochondrial dysfunction is not observed in heterozygous GBA knockout neurons [262] PD-associated GBA variants may convey specific gain-of-function effects in neurons, aside from lysosomal function. Due to the implications of GBA-associated lysosomal dysfunction and PD-associated pathologies, GluCer synthesis and metabolism have become promising targets for therapeutic intervention [254, 263–265], as have molecules such as ubiquitin ligase NEDD4, which target alpha-synuclein for lysosomal destruction [266].

Lysosomal dysfunction can also contribute to neurodegeneration through mutations that do not directly affect hydrolytic enzymes. Niemann-Pick disease type C (NPC) is a juvenile onset neurodegenerative condition with death occurring in young adulthood, primarily effecting the cerebellum, associated with accumulation of a range of lipids within the lysosomal lumen including cholesterol, sphingomyelin and sphingosine [267–271]. The disorder has two genetically distinct forms; NPC1 is a sterol-sensing transmembrane protein acidic compartments [50, 51, 272] and rarer mutations in NPC2 that disrupt a lysosomal soluble peptide with a cholesterol binding domain [52, 53, 273]. As NPC1 is expressed in most cell types, why neurons are particularly vulnerable to its dysfunction is unclear. Experimental data has suggested defective regulation of lysosomal calcium may contribute to NPC associated phenotypes, with increased storage of lysosomal sphingosine causing a reduction in luminal calcium levels, subsequent accumulation of further lipids and defects in endocytic trafficking [274]. Since NPC shares formation of the hyperphosphorylated Tau neurofibrillary tangles typically seen in AD and PD [275], understanding the mechanistic role of NPC1/2 in lysosome function may have broader implications for other neurodegenerative diseases and their treatment.

### Lysosome positioning

The positioning of lysosomes within a cell is intertwined with the function of these vesicles, particularly with regard to acidification of the lumen. In non-polarised cells, lysosomes are distributed into two groups; a relatively stationary perinuclear “cloud” [276] where early endosomes mature through to lysosomes, and a highly motile population in the periphery [277]. Lysosome transportation to the periphery generally occurs along microtubule networks, with anterograde transport to the periphery mediated by kinesin motor proteins [278], and returning retrograde transport by the dynactin motor complex [279]. Lysosome distribution differs somewhat in highly polarised neurons, where the vast length and volume of many axons requires effective delivery of acidified lysosomes. Though lysosomes can be detected throughout the soma, axon, dendrites and synapses of neurons, their positioning appears to define their function. Mature, acidified lysosomes are enriched in the soma, with a decreasing gradient of acidity along the distal-proximal length of the axon, suggesting degradation within lysosomes occurs in the cell body [280, 281]. Directionality of lysosome transport within the axon has not been fully resolved, in part due to differences in the assays used for their detection [282]. Further research into the basic neurobiology of lysosome maintenance and trafficking is long overdue and would enable us to better understand neurodegenerative disease.

Abnormal transport and positioning of lysosomes may contribute towards the pathogenesis of AD, particularly as disruption of the endo-lysosomal system is one of the earliest detectable histopathological features [206]. Swollen, dystrophic neurites are a common histopathological feature of AD [283, 284], with lysosomes and related vesicles found to accumulate within these axonal swellings. Curiously, such lysosome enriched swellings are often in regions proximal to amyloid plaques in patient brains [285–287] and rodent models of the disease [288]. Whether accumulation of lysosomes cause amyloid pathology, or a secondary event downstream of plaque formation, remains unanswered. It is conceivable that plaques and the neuroinflammation may alter local intra-axonal processes such as transportation, however emerging experimental data suggests that dysregulation of lysosome axonal transport may actively drive amyloid accumulation. Proteins in the c-Jun N-terminal kinase-interacting proteins (JIP) family of conserved mitogen activated protein kinases (MAPKs) regulate microtubule mediated transport of cargos along axons [289, 290]. Mutation of JIP3 causes accumulation of lysosomal vesicles, amyloid processing enzymes and increased production of toxic species of Aβ in an AD mouse model [291]. Further links between AD pathology and lysosome function can be found in the function of PSEN proteins. Early onset AD patients with PSEN1 or 2 mutations present elevated lysosomal pathology levels or lysosome associated pathology [292]. Experimental disruption of PSEN1 in cell culture models results in reduced assembly of the vacuolar-type H+ ATPase (vATPase) complex at the lysosomal membrane and subsequent failure of the lumen to reach correct acidic pH [293]. Consequentially, lysosomal acid-sensitive hydrolytic enzymes have reduced function and increased efflux of luminal Ca^2+^. Taken together, evidence of accumulation of axonal lysosomes, increasing amyloid plaque burden and lysosomal dysfunction associated hereditary AD genes are suggestive that impaired lysosomal positioning may be a contributing factor in AD that warrants further investigation. Furthermore, as mislocalisation of lysosomes has also been reported in cellular models of HD [294] and ALS associated mutant dynactin-p150^glued^ [295], disruption of their trafficking may be a common pathogenic event in neurodegeneration.

## Conclusions and future perspectives

Changes in autophagy, mitophagy and endo-lysosomal processes have been implicated in most neurodegenerative diseases, however their contribution is still only partially defined, with several outstanding questions. Though the regulatory processes underpinning autophagy are well understood with regard to starvation, its regulation in neurons in health and disease is poorly defined. The initiating signals for upregulation of autophagy in times of neurotoxic stress are not well understood, particularly how the right balance between homeostatic autophagy and the clearance of toxic material is achieved. More so, it is not entirely clear if autophagy is indeed protective, or is instead contributing to neuronal stress and destruction. This is in part due to gaps in our basic understanding of the cellular mechanism driving autophagy. Though a peripheral origin and retrograde transport of autophagosomes has been demonstrated in tissue culture experiments [67], the source of the phagophore isolation membrane in neurons is not clear and requires further description in vivo. Experiments in *Drosophila* have also suggested an important role for microautophagy in the maintenance of synapses [122], a highly vulnerable neuronal compartment, and this process warrants further investigation in the context of neurodegenerative disease.

Despite extensive exploration of the function of PINK1 and PRKN in Parkinsonisms, robust evidence for defective mitophagy as a direct cause of pathology in patient brains is lacking. It is clear that PINK1 and PRKN have independent roles outside of mitophagy [296, 297], and furthermore, there is evidence to suggest that basal mitophagy can occur independently of PINK1 or PRKN [298, 299]. The importance of PINK1/PRKN mediated mitophagy to the viability of dopaminergic neurons in the substantia nigra thus needs to be clarified. The link between mitophagy and neuroinflammation is not well characterised, but new findings indicate that the metabolic state of microglia influence their activation [162] and this can be regulated by mitochondrial turnover [159]. The relationship between mitochondrial quality control in glial cells and neurodegenerative disorders may reflect the convergence of two key processes in neurodegenerative disorders and therefore requires further investigation.

It is curious why specific mechanisms within the endosomal pathway appear to be dysregulated in different neurological conditions, suggesting divergent pathological roles in neurodegeneration. While EE dysfunction appears to be a characteristic feature of multiple neurodegenerative diseases, RE is primarily implicated in HD and AD, whereas LE pathway dysfunction is largely restricted to PD. Disruption of synaptic receptor recycling, observed when the RE is compromised [231], may be of a significant importance to medium spiny neurons and cortical/hippocampal neurons that underpin learning and memory through spine remodelling. The LE pathway may on the other hand play a more prominent feature in dopaminergic neurons, where the disposal of mitochondria and alpha-synuclein is prioritised [156, 300]. It is currently unclear why EE dysfunction appears to be a pathological feature of many different diseases. Does EE dysfunction always lead to lysosomal problems downstream or is the LE system adaptable enough to correct itself despite endocytosis and sorting issues? Cumulative evidence supports the latter, suggesting that lack of LE-lysosome fusion can to some degree be compensated for by autophagic clearance. The endosomal pathway is a dynamic continuum and a shift in its balance may result in neuronal demise, as evidenced by both causative and enhanced disease risk associated mutations.

Finally, abundant data suggests that defects in autophagy and the endo-lysosomal system contribute to disease, supporting the concept that their stimulation is a feasible target for therapeutic intervention in neurodegeneration. Several pharmacological modifiers of autophagy with blood brain barrier permeable properties exist, with some experimental evidence to support their use [98, 99, 301, 302]. These are generally not considered appropriate for long-term use due to global alterations of essential cellular processes [303]. Identifying potent, neuro-specific modulators of autophagy and endo-lysosomal function will be essential to determine if these pathways are truly viable targets for therapeutics, in order to ultimately treat devastating neurodegenerative disorders.

## Data Availability

All work cited is in the public domain.

## Abbreviations

AD: Alzheimer’s disease
AIM/LIR: ATG8 Interaction motif/LC3 interacting region
ALS: Amyotrophic Lateral Sclerosis
ALS2: Alsin Rho Guanine Nucleotide Exchange Factor
AMPK: 5′- Adenosine Monophosphate-activated Protein Kinase
APOE4: Apolipoprotein E4
APP: Amyloid Precursor Protein
ARJP: Autosomal-Recessive Juvenile Parkinsonism
ATG: Autophagy Related Gene
Aβ: Amyloid Beta
BACE: Beta-Secretase 1
BECN1: Beclin1
BIN1: Bridging Integrator 1
C9ORF72: Chromosome 9 Open Reading Frame 72
cAMP: Cyclic Adenosine Monophosphate
CHMP2B: Charged Multi-Vesicular Body Protein 2B
CMA: Chaperone-Mediated Autophagy
DJ-1: Parkinsonism Associated Deglycase
DNAJC: DnaJ/Heat Shock Protein Family (HSP40) co-chaperone
EE: Early Endosome
EEA1: Early Endosome Antigen 1
EGFR: Epidermal Growth Factor Receptor
ER: Endoplasmic Reticulum
ESCRT: Endosomal Sorting Complex Required for Transport
FAM134B: Family with sequence similarity 134, Member B
FBXO7: F-Box Only Protein 7
FIP200: Focal Adhesion Kinase Family Kinase-Interacting Protein Of 200KDa
FTD: Frontotemporal Dementia
GAK: Cyclin G Associated Kinase
GBA: β-glucocerebrosidase
GDP: Guanosine Diphosphate
GEF: Guanine Nucleotide Exchange Factor
GluCer: Glucosylceramide
GTP: Guanosine Triphosphate
GWAS: Genome Wide Association Study
HAP40: HTT associated protein 40
HD: Huntington’s disease
HSC70: Heat Shock Protein Family A (Hsp70) Member 8
HSP90: Heat Shock Protein 90
HTT: Huntingtin
iPSC: induced Pluripotent Stem Cell
JIP: c-Jun N-terminal kinase-interacting proteins
LAMP2A: Lysosome-Associated Membrane Protein 2A
LC3-I: Microtubule Associated Protein 1 Light Chain 3 – non-lipidated
LC3-II: Microtubule Associated Protein 1 Light Chain 3 - phosphatidylethanolamine lipidated
LE: Late Endosome
LRRK2: Leucine Rich Repeat Kinase 2
MAPK: Mitogen Activated Protein Kinases
MEF2D: Myocyte Enhancer Factor 2D
MFN1: Mitofusin 1
MOM: Mitochondrial Outer Membrane
mTOR: mammalian Target Of Rapamycin
mTORC1: mammalian Target Of Rapamycin Complex 1
MVB: Multi-Vesicular Body
NADH: Nicotinamide adenine dinucleotide
NBR1: Neighbour Of BRCA1 Gene 1 Autophagy Cargo Receptor
NCL: Neuronal ceroid lipofuscinosis
NDP52: Nuclear Domain 10 Protein 52/ Calcium Binding And Coiled-Coil Domain 2
NEDD4: Neural Precursor Cell Expressed, Developmentally Down-Regulated 4
NPC: Niemann-Pick disease type C
OPTN: Optineurin
p62: Phosphotyrosine-Independent Ligand For The Lck SH2 Domain Of 62 KDa / Sequestosome-1
PARL: Presenilin Associated Rhomboid-Like
PD: Parkinson’s disease
PI(3)P: Phosphatidylinositol 3-phosphate
PINK1: PTEN-Induced Kinase 1
poly-Q: poly-Glutamine
PRKN: Parkin RBR E3 Ubiquitin Protein Ligase
PSEN: Presenlin
RAB: GTPase RAB guanosine triphophatase
RE: Recycling Endosome
Rheb: RAS homolog enriched in the brain
Rhes: RAS homolog enriched in the striatum
RIN: RAS and RAB interactor 1
SH3TC2: SH3 domain and tetratricopeptide repeats 2
SNARE: Soluble N-ethylmaleimide-sensitive fusion protein attachment protein Receptor
SNX: Sorting Nexin
SORCS1: Sortilin Related VPS10 Domain Containing Receptor 1
SORL1: Sortilin Related Receptor 1
SYNJ1: Synaptojanin 1
TAX1BP1: TAX1 Binding Protein 1
TBK1: TANK Binding Kinase 1
UA: Urolithin A
UCHL-1: Ubiquitin carboxyl-terminal esterase L1
ULK1: Unc-51-like Autophagy Activating Kinase 1
vATPase: vacuolar-Type H^+^ ATPase
VPS: Vacuolar Protein Sorting
